# Obesity associated cancers, genetics, epigenetics and elephants

**DOI:** 10.20517/jtgg.2023.23

**Published:** 2023-08-24

**Authors:** Nathan A. Berger

**Affiliations:** Case Comprehensive Cancer Center, School of Medicine, Case Western Reserve University, Cleveland, OH 44106, USA.

Obesity is estimated to affect more than 110 million children and 640 million adults and is associated with significant co-morbidities, including cardiovascular diseases, Type II diabetes mellitus, and thirteen types of cancer^[1]^. *JTGG* recently published a special edition focused on Genetics and Epigenetics of Obesity Associated Cancers to help better understand factors driving the obesity - cancer linkage.

Qu *et al.* reported that, compared to low-fat diet, when subjecting the C57Bl6J APC^+/+^ and C57Bl6J APC^Min/+^ mice to high-fat diet for only 3 days, there was a noticeable shift in the acetylation of Lysine 27 in Histone H3 (H3K27ac), an epigenetic determinant of transcriptionally active chromatin, favoring fatty acid metabolism in support of accelerated growth while simultaneously suppressing immunologic mechanisms that could potentially contribute to the control of tumor growth^[2]^. Not all transcriptional changes were associated with H3K27 acetylation, suggesting the potential involvement of other epigenetic mechanisms. Interestingly, at the early time point of 3 days on high-fat diet, there were epigenetic signs indicating activation of Wnt signaling, but no signs by RNA Seq that this pathway was upregulated^[2]^.

Using similar techniques to measure H3K27ac, Li *et al.* showed that 15–20 weeks of high-fat diet feeding to C57Bl6 mice reproduced the chromatin gene enhancer landscape to resemble that in colorectal cancer^[3]^. They showed that genes dysregulated by high-fat diet were connected to inflammation, cancer, cellular movement, and tissue development. When similar studies were conducted in NAG-1 transgenic mice, which are resistant to high-fat-diet-induced weight gain, the chromatin changes characteristic of cancer were not identified. The authors concluded that the pro-carcinogenic epigenetic changes were due to obesity rather than diet itself[3].

Exploration of DNA methylation in high-fat diet-fed C57Bl6J mice showed epigenetic changes supportive of long-chain fatty acid oxidation and downregulation of feedback inhibitors of pro-proliferative signaling pathways in older mice[4]. Obesity-associated upregulated genes in aged mice were associated with cell cycle, DNA replication and repair, and chromatin organization. It is noteworthy that these changes were reversible, following long-term (28 weeks) but not short-term (5 weeks) weight loss[4].

Focusing their analysis on the proteome, Comertpay and Gov[5] applied a machine learning approach to a high-throughput gene expression dataset from patients with breast cancer to study obesity-associated protein networks, interactions and differentially expressed genes. They identified 19 downregulated and 4 upregulated genes in obese compared to non-obese women with breast cancer, indicating significant activation of cancer-associated pathways, including RAF-independent MAPK 1/3 activation, collagen degradation, bladder cancer, cytochrome P450 metabolism, and hedgehog. These observations may be useful for risk assessment of disease progression in patients with breast cancer and approaches to precision medicine[5].

In a tri-racial ethnic population of breast cancer patients, Puyana *et al.* used 35 externally validated, single nucleotide polymorphisms to determine whether racial/ethnic differences in Polygenic Risk Score (PRS) contribute to obesity and inflammatory biomarkers in patients with breast cancer[6]. They identified an obesity PRS that correlated with body mass index and CRP levels that were associated with eligibility for bariatric surgery. Although little is known about the impact of bariatric - metabolic surgery on obesity-influenced outcomes in patients with breast cancer, the authors suggested that PRS could be employed to inform the application of bariatric - metabolic surgery in patients with breast cancer to improve obesity-related effects on recurrence, metastasis, and mortality[6].

These reports provide insights into selected aspects of the broad spectrum of genetic, epigenetic, and proteomic data available for obesity-associated cancers that may be potentially useful as targets for therapeutic intervention and/or as biomarkers for monitoring cancer predisposition, prognosis, and preventive strategies. At the same time, their diversity is somewhat reminiscent of the Buddhist parable of the group of blind men asked to describe an elephant by touching different parts to examine the animal. Each man described the elephant based on their limited experience: leg, trunk, ear, and tusk, but no one could provide a comprehensive description of the entire animal. Practically speaking, no one would know how to deal with the elephant based solely on their limited encounters. By analogy to the parable, what is clearly needed is a coordinated, comprehensive, multi-omic assessment of obesity-associated cancer, genetics, epigenetics, and proteomic characteristics over a time course to gain a deeper understanding of how these factors influence cancer incidence, progression, and control, and to shed light on potential therapeutic targets within these different aspects. In addition, more detailed information on the genetic, epigenetic and proteomic consequences of exercise, bariatric - metabolic surgery and new classes of weight loss pharmaceutical agents will further enhance our comprehension and strategies pertaining to obesity-associated cancers. And by the way, what do we know about genetics, epigenetics and proteomics of obesity associated cancers in the elephant, the largest land mammal, with the greatest fat mass.

## Data Availability

Not applicable.
